# Prophylactic Subacute Administration of Zinc Increases CCL2, CCR2, FGF2, and IGF-1 Expression and Prevents the Long-Term Memory Loss in a Rat Model of Cerebral Hypoxia-Ischemia

**DOI:** 10.1155/2015/375391

**Published:** 2015-08-18

**Authors:** Victor Manuel Blanco-Alvarez, Guadalupe Soto-Rodriguez, Juan Antonio Gonzalez-Barrios, Daniel Martinez-Fong, Eduardo Brambila, Maricela Torres-Soto, Ana Karina Aguilar-Peralta, Alejandro Gonzalez-Vazquez, Constantino Tomás-Sanchez, I. Daniel Limón, Jose R. Eguibar, Araceli Ugarte, Jeanett Hernandez-Castillo, Bertha Alicia Leon-Chavez

**Affiliations:** ^1^Facultad de Ciencias Químicas, BUAP, 14 Sur y Avenida San Claudio, 72570 Puebla, PUE, Mexico; ^2^Laboratorio de Medicina Genómica, Hospital Regional 1° de Octubre, ISSSTE, Avenida Instituto Politécnico Nacional No. 1669, 07760 México, DF, Mexico; ^3^Departamento de Fisiología, Biofísica y Neurociencias, Centro de Investigación y de Estudios Avanzados del Instituto Politécnico Nacional, Apartado Postal 14-740, 07000 México, DF, Mexico; ^4^Instituto de Fisiología, BUAP, 14 Sur 6301, 72570 Puebla, PUE, Mexico

## Abstract

Prophylactic subacute administration of zinc decreases lipoperoxidation and cell death following a transient cerebral hypoxia-ischemia, thus suggesting neuroprotective and preconditioning effects. Chemokines and growth factors are also involved in the neuroprotective effect in hypoxia-ischemia. We explored whether zinc prevents the cerebral cortex-hippocampus injury through regulation of CCL2, CCR2, FGF2, and IGF-1 expression following a 10 min of common carotid artery occlusion (CCAO). Male rats were grouped as follows: (1) Zn96h, rats injected with ZnCl_2_ (one dose every 24 h during four days); (2) Zn96h + CCAO, rats treated with ZnCl_2_ before CCAO; (3) CCAO, rats with CCAO only; (4) Sham group, rats with mock CCAO; and (5) untreated rats. The cerebral cortex-hippocampus was dissected at different times before and after CCAO. CCL2/CCR2, FGF2, and IGF-1 expression was assessed by RT-PCR and ELISA. Learning in Morris Water Maze was achieved by daily training during 5 days. Long-term memory was evaluated on day 7 after learning. Subacute administration of zinc increased expression of CCL2, CCR2, FGF2, and IGF-1 in the early and late phases of postreperfusion and prevented the CCAO-induced memory loss in the rat. These results might be explained by the induction of neural plasticity because of the expression of CCL2 and growth factors.

## 1. Introduction

The protective role of zinc in cerebral ischemia has been clearly sustained [1–3]. Several studies have shown that the ischemic preconditioning and postconditioning decrease intracellular zinc accumulation in a gerbil model of oxygen-glucose deprivation, thus causing ischemic tolerance [4, 5]. Interestingly, an increase in zinc at sublethal levels has also an effect similar to that of the ischemic preconditioning [6], involving caspase-3 activation, PARP-1 cleavage, and HSP70 induction, all of which are crucial for subsequent neuroprotection against glutamate excitotoxicity [2] and zinc toxicity [7, 8]. An administration of zinc, protoporphyrin, superoxide dismutase Zn-Cu (SOD), or PEP-SOD1 triggers protective mechanisms in different animal models and patients with cerebrovascular disease [9–12]. Some of these mechanisms are induction of metallothioneins, increase in the antioxidant capacity, increase in the growth factors such as GH, IGF1, and IGFBP3 [13, 14], and a decrease in the iron-catalyzed lipid peroxidation [1, 15, 16]. In addition, zinc participates in neural plasticity, promoting glutamate release and neuronal excitability in the central nervous system [17–19]. All these mechanisms lead to a decrease in inflammation and cell death [1, 3, 12, 16]. Specifically in case of inflammation, zinc decreases the level of cytokines [16], importantly CCL2 and CCL3 [20].

In the cerebral ischemia/reperfusion-induced injury, proinflammatory cytokines and chemokines are rapidly upregulated. For instance, increased serum level of chemokines such as CCL2 (monocyte chemoattractant protein-1), CCL5, and CXCL1 have been detected in the earlier days after ischemia [21–23]. During hypoxia-ischemia, the expression of CCL2 is known to be stimulated by the hypoxia-inducible factor-1 (HIF-1) in astrocytes [24] and neurons [25]. Current evidence points out that the increased level of CCL2, CCL5, and CXCL1 during ischemia plays a dual role and could be either harmful or beneficial. This work is focused on CCL2 because it is one of the main chemokines that plays a major role in promoting leukocyte infiltration into the brain parenchyma during ischemia-induced inflammatory response [26, 27] and neuroregeneration including angiogenesis, neurogenesis, and synaptic plasticity [28–34].

The harmful effect of CCL2 has been related to its overexpression in astrocytes, which causes delayed death of the pyramidal neurons after ischemia [35]. The postulated mechanism is that the CCL2 favors the infiltration of macrophages and several leukocyte subtypes to the brain [27, 36, 37] that produce neuroinflammation by increasing the permeability of brain endothelial blood-brain barrier [27, 38]. In support of the harmful effect, experiments in CCR2 (−/−) mice have concluded that the absence of the CCL2 receptor (CCR2) prevents the cerebral injury following ischemia/reperfusion [39].

The beneficial effect of CCL2 also is sustained by several experimental evidences. Hypoxic preconditioning induced CCL2 upregulation has been shown to participate in the ischemic tolerance [40, 41]. Similarly, CCR2 upregulation induced by either ischemic preconditioning or ischemic postconditioning also markedly prevents ischemia/reperfusion-induced cerebral injury as measured in terms of infarct size, loss of memory, and motor coordination [41]. The CCL2/CCR2 interaction also stimulates the chemotaxis of neural stem cells (NSCs) to the ischemic zone in the brain from the neurogenic niches as a compensatory mechanism to repair damaged brain after stroke [31]. A neurogenic role has also been attributed to insulin-like growth factor-1 (IGF-1) and CXCL12/SDF-1, which exert a considerable regulation on proliferation, migration, and survival of NSCs [42]. Cerebral mRNA expression of IGF1, FGF2, TGF, EGF, and PDGF-A has been observed in ischemic preconditioning [43, 44]. Furthermore, administration of IGF-1 and FGF2 has been effective in preventing the ischemic stroke possibly promoting neuronal plasticity [45–50]. For instance, chronically elevated levels of CCL2 in the hippocampus produce hippocampal synaptic plasticity that block the depressing effects of ethanol [34].

In the current work, we hypothesized that the subacute administration of zinc (2.5 mg/kg ZnCl_2_ every 24 h for 4 days) will exert an ischemic-like preconditioning and produce an increase in the expression of CCL2, CCR2, IGF-1, and FGF2 after a transient common carotid artery occlusion (CCAO) for 10 min. Because estrogen and testosterone have been shown to be neuroprotective against ischemic insult [51, 52] and estrogen increases zinc levels in the brain [53], we used male rats to test our hypothesis. To the best of our knowledge, testosterone has not been related to increased levels of zinc in the brain. The beneficial effect of the subacute zinc administration will be reflected by the prevention of long-term memory loss that is induced by CCAO. RT-PCR and ELISA were used to illustrate the expression of CCL2, CCR2, IGF-1, and FGF2 in the cerebral cortex-hippocampus. CCR2 was also evaluated by immunofluorescence in slices of those regions. The Morris Water Maze was used to assess whether the subacute administration of zinc prevents the CCAO-induced loss of spatial reference memory. Our results suggest that the subacute administration of zinc has a protective effect in an animal model of cerebral-vascular disease.

## 2. Materials and Methods

### 2.1. Experimental Animals

Male Wistar rats between 190 g and 240 g (2 to 2.5 months old) were obtained from the vivarium of the CINVESTAV. Animals were maintained in adequate rooms with controlled conditions for temperature (22 ± 3°C) and a light-dark cycle (12 h-12 h; light onset at 0700). Food and water were provided* ad libitum*. All procedures were in accordance with the Mexican current legislation, the NOM-062-ZOO-1999 (SAGARPA), based on the Guide for the Care and Use of Laboratory Animals, NRC. The Institutional Animal Care and Use Committee approved the experimental procedures with the protocol number 09-102. All efforts were made to minimize animal suffering.

### 2.2. Zinc Administration

Rats were grouped according to different treatments: (1) Zn96h, control rats treated with a subacute administration of zinc (ZnCl_2_; 2.5 mg/kg every 24 h for 4 days). Brains were obtained at 24, 48, 72, and 96 h during zinc administration, and at 4, 8, 12, 24, 36, 96, and 168 h after administration. (2) Zn96h + CCAO, rats were treated with a subacute administration of zinc and subjected to transient ischemia through a common carotid artery occlusion (CCAO) for 10 min 24 h after the last administration of zinc. (3) CCAO, rats with CCAO only. (4) Sham group, rats with mock CCAO. (5) Control, rats without any treatment. Brains were obtained at different hours (4, 8, 12, 24, 36, 96, and 168 h after reperfusion) from animals of groups 2 to 4. Brains obtained from control rats were considered as time −96 h.

### 2.3. Enzyme-Linked Immunosorbent Assay (ELISA)

CCL2, CCR2, FGF2, and IGF-1 levels were measured by ELISA in homogenates of temporoparietal cortex-hippocampus (*n* = 5 for each group), as described previously [54]. Protein content was determined using the method by Sedmak and Grossberg [55]. Aliquots containing 5 *μ*g of total protein were placed into wells of ELISA plates. Subsequently, 100 *μ*L of 0.1 M carbonate buffer was added into each well and the plates were incubated at 4°C for 18 h. To block nonspecific binding sites, 200 *μ*L of 0.5% bovine serum albumin (IgG free) was added to each well at room temperature (RT). After 30 min of incubation, the wells were washed thrice with phosphate buffered saline- (PBS-) Tween 20 (0.1%). Rabbit monoclonal antibodies to CCL2 (1 : 500 dilution; Cat. # ab7202), CCR2 (1 : 500 dilution; Cat. # ab21667), and FGF2 (1 : 500 dilution; Cat. # ab106245) and mouse monoclonal antibody to IGF-1 (1 : 500 dilution; Cat. # ab36532) were added into each well and incubated for 2 h at RT. All the antibodies were purchased from Abcam (Cambridge, MA, USA). After three washes with PBS, a horseradish-peroxidase conjugated goat anti-rabbit or mouse IgG (1 : 1000 dilution; Dako North America Inc; Carpinteria, CA, USA) was added into the wells and incubated for 2 h at RT. The antibody-antigen complex was revealed by adding 100 *μ*L of 2,2′-azino-bis(3-ethylbenzthiazoline-6-sulphonic acid) (ABTS) containing 0.3% H_2_O_2_ into each well. After 15 min, optical density (OD) was determined using a Benchmark multiplate reader at 415 nm (Bio-Rad, Hercules, CA, USA) as described elsewhere [54]. All samples were made under the same experimental conditions and time.

### 2.4. Immunolabeling of CCR2

CCR2 was detected by indirect immunofluorescence techniques in coronal brain slices of Wistar rats (*n* = 3 in each group). Rats were deeply anesthetized with chloral hydrate and were perfused through the ascending aorta with 100 mL of PBS followed by 150 mL of 4% paraformaldehyde in PBS. Brains were then removed and maintained in a fixative at 4°C for 48 h. Each brain was embedded in paraffin and then sectioned into 3 *μ*m slices on the sagittal plane using a Leica RM 2135 microtome (Leica Microsystems; Nussloch, Germany). Slices were individually collected on a glass slide. Tissue slices, previously deparaffinized, were rehydrated and incubated with 0.5% IgG-free bovine serum albumin in PBS-Tween 20 (0.1%) for 20 min at room temperature. The primary antibody was the rabbit monoclonal anti-CCR2 (1 : 200 dilution; cat. # ab32144, Abcam; Cambridge, MA, USA). The secondary antibody was a fluorescein (FITC) goat anti-rabbit IgG (1 : 60 dilution; Millipore; Temecula, CA, USA). The counterstaining was made using propidium iodide (2 *μ*g/mL). Tissue slices were mounted on glass slides using Vectashield (Vector Laboratories; Burlington; Ontario, Canada). The fluorescence within the cells was analyzed with 5x and 40x objectives of a Leica DMIRE2 microscope using the filters K3 for FITC and TX2 for propidium iodide (Leica Microsystems; Wetzlar, Germany). Images were digitalized with a Leica DC300F camera (Leica Microsystems; Nussloch, Germany) and processed with a workstation Leica FW4000, version V1.2.1 (Leica Microsystems Vertrieb GmbH; Bensheim, Germany).

### 2.5. Reverse Transcriptase-Polymerase Chain Reaction

The RT-PCR technique was used to determine mRNA levels of the CCL2, CCR2, FGF2, IGF-1, and glyceraldehyde-3-phosphate dehydrogenase (GA3PDH) as housekeeping gene in homogenates of temporoparietal cortex-hippocampus of controls and experimental groups (*n* = 5 for each group) as described elsewhere [54, 56]. mRNA was extracted from 100 mg of tissue (temporoparietal cortex and hippocampus) in 1 mL of TRizol reagent (Invitrogen, Life Technologies; Carlsbad, CA, USA), quantified by spectrophotometry at 260 nm, and analyzed by using 1% agarose gel electrophoresis. Total RNA (5 *μ*g) was transcribed by using SuperScript III reverse transcriptase (200 U) and 0.1 *μ*g of poli-T primer (Invitrogen; Carlsbad, CA; USA). Two *μ*L of cDNA diluted 1 : 4 was amplified in a thermocycler (Gene Amp PCR System 9700; Applied Biosystems; Foster city, CA, USA) using 0.2 *μ*M of each sense and antisense primer (Table 1) and 2.5 U of platinum Taq DNA polymerase in a final volume of 25 *μ*L. After an initial denaturation at 94°C for 5 min, amplification was made using 36 cycles as follows: denaturation, 94°C for 40 s (CCL2, CCR2, FGF2, IGF-1, and G3PDH); annealing, 57°C (CCL2, CCR2, FGF2, and IGF-1) and 55°C (GA3PDH) for 30 s; and extension, 72°C for 20 s (CCR2, FGF2, IGF-1, and GA3PDH) and 10 s (CCL2). The PCR products were analyzed by using electrophoresis on 2% agarose gel prestained with ethidium bromide (0.5 *μ*g/mL). Upon completion of electrophoresis, PCR products were photographed with a Kodak MI photodocumentation system (Mod. Gel Logic 200-E2000 V 5.0.1.30; Eastman Kodak Co; Rochester, NY, USA). Densitometric analysis was accomplished using the software Kodak MI (Eastman Kodak Co; Rochester, NY, USA). The densitometric values (arbitrary units) of the bands were normalized with respect to the densitometric values of GA3PDH band and with respect to the values of control without treatment. Expected size amplicons were cut from the gel and purified in the Zymo Spin columns (Zymo Research, CA 92614, USA). Posteriorly, all amplicons were sequenced by using capillary technology and detection system of fluorescence induced by laser in an automatic sequencer (Beckman Coulter, model CEQ 2000XL, Saint-Julie, Quebec, Canada).

### 2.6. Spatial Reference Learning and Memory

The Morris Water Maze was used to measure the spatial reference memory. The measurements were conducted in a round tank, 150 cm in diameter and 80 cm deep, filled with water and divided into four imaginary quadrants. Water was maintained at a temperature of 23 ± 2°C and dyed white with a titanium dioxide suspension to prevent the rats from locating the platform visually. Several distal visual cues were placed on both the walls of the Morris Water Maze and the room in which it had been installed. This evaluation consisted of five test days with four consecutive trials per day. During the trial, each animal was left in the tank facing the wall and allowed to swim freely to an escape platform (40 cm in height and 15 cm in diameter), which was submerged by 2 cm under the water surface and conserved to the center of southeast quadrant of the tank. Rats were left in the tank on each of the four vertices of the imaginary quadrants. If the animals did not find the platform during a period of 60 s in the first trial of each test day, they were gently guided to it, allowed to remain on the platform for 30 s, and removed from the tank for 30 s. This procedure was used to ensure that the animals retained the visuospatial information of the maze online during the execution of the swimming task [57]. Long-term memory was evaluated in the absence of the platform on day 7 after learning. The latency to reach the platform and the number of times that rats pass by platform location were measured.

### 2.7. Statistical Analysis

The results for the difference between mRNA levels in various aged groups are expressed as mean ± SEM. All assays were analyzed by using a one-way ANOVA test. Post hoc Dunnett's test was used to determine difference between the control group (without treatment) and treatment groups. The differences between the sham and control treated with zinc versus experimental groups (CCAO or Zn96h + CCAO) were determined using unpaired Student's *t*-test. The transcription change expressed in optical density (Fold change) was assessed by normalizing the sample test against GAD3PDH and untreated control. The normalized values for ELISAs are expressed as the sample test against untreated control. All statistical analyses were performed using the GraphPad Prism software (GraphPad Software Inc.; San Diego, CA, USA). *P* values < 0.05 were considered significant.

## 3. Results

**Figure 1:**
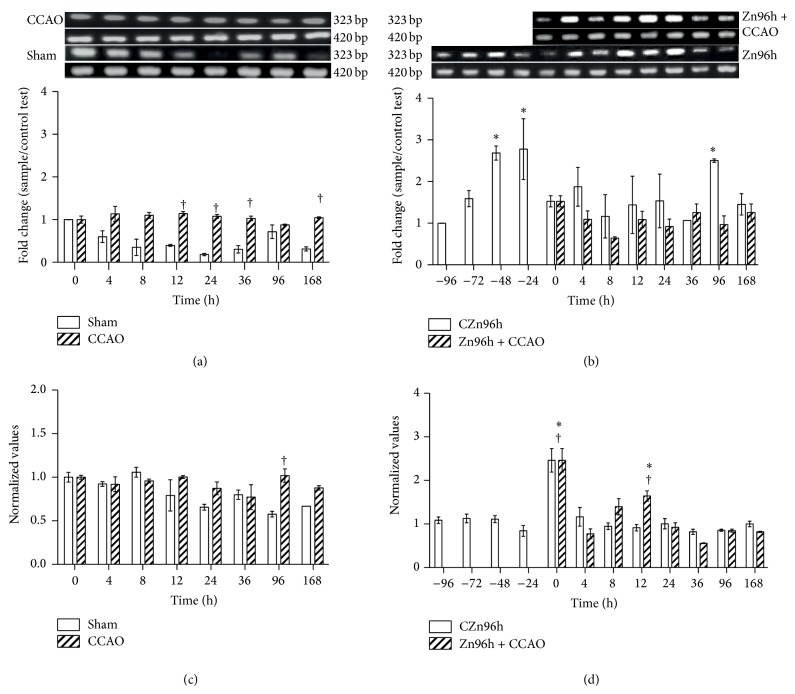
Effect of subacute administration of zinc on CCL2 expression in cerebral cortex-hippocampus of the rat. RT-PCR was used to determine mRNA levels of CCL2 (323 bp) and GA3PDH (420 bp). ((a) and (b)) Showing representative photographs of ethidium-bromide-stained RT-PCR products fractionated on 2% agarose gel and the respective densitometry analysis. ((c) and (d)) Showing the CCL2 protein levels using ELISA. Each value represents the mean ± SEM of 5 independent experiments made in triplicate. (1) Zn96h, rats injected with ZnCl_2_ (one dose every 24 h during 4 days). (2) Zn96h + CCAO, rats treated with zinc before 10 min of common carotid artery occlusion (CCAO). (3) CCAO, rats with CCAO only. (4) Sham group, rats with mock CCAO. (5) Untreated rats. *∗*, significant when compared with the control group; ANOVA test and post hoc Dunnett's test. †, significant when compared between groups; unpaired Student's *t*-test. *P* < 0.05.

Protein and mRNA levels of CCL2, CCR2, FGF2, and IGF-1 were determined in the cerebral cortex-hippocampus to find out whether a subacute administration of zinc causes a preconditioning effect in CCAO model. Our results showed that 10 min CCAO did not modify the levels of CCL2 mRNA (Figure 1(a)) and protein (Figure 1(c)) after reperfusion when compared with the untreated control (time 0). A statistically significant difference was observed when mRNA values in the CCAO group were compared with the sham group at 12 h, 24 h, 36 h, and 168 h (Figure 1(a)) and for protein at 96 h (Figure 1(c)). The subacute administration of zinc produced upregulation of CCL2 mRNA by 169%  ±  17% at 48 h and 178%  ±  73% at 24 h before time 0 and by 151%  ±  4% at 96 h after time 0 (Figure 1(b)). After CCAO, no change in the level of CCL2 was observed in rats treated with zinc (Figure 1(b)). The CCL2 protein levels increased by 146%  ±  27% at time 0 and by 64%  ±  12% at 12 h after reperfusion in rats treated with zinc (Figure 1(d)).

**Figure 2:**
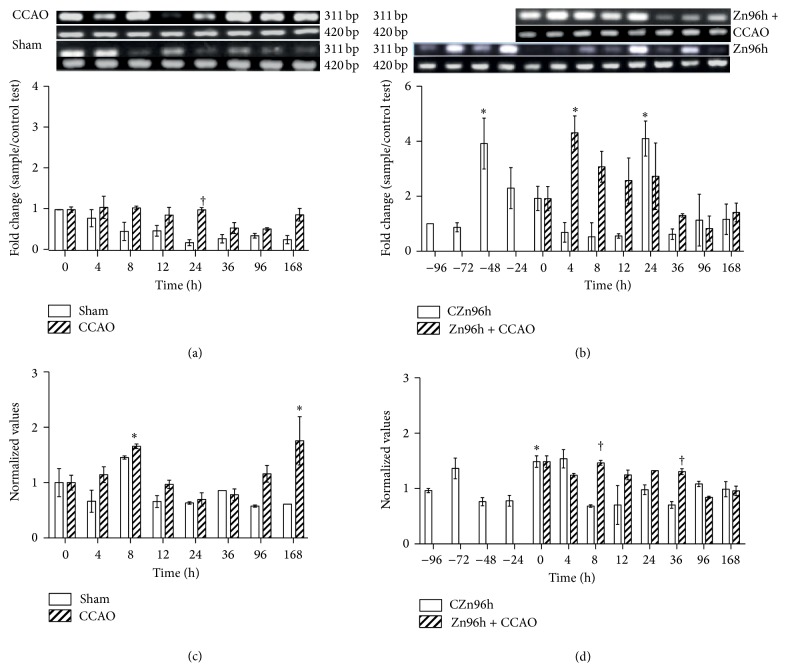
The subacute administration effect of zinc on levels of CCR2 expression after hypoxia-ischemia in rats. RT-PCR was used to determine mRNA levels of CCR2 (311 bp) and GA3PDH (420 bp). ((a) and (b)) Showing representative photographs of ethidium-bromide-stained RT-PCR products fractionated on 2% agarose gel and the respective densitometry analysis. ((c) and (d)) Showing the CCR2 protein levels measured using ELISA. Each value represents the mean ± SEM of 5 independent experiments in triplicate. (1) Zn96h, rats injected with ZnCl_2_ (one dose every 24 h during 4 days). (2) Zn96h + CCAO, rats treated with zinc before 10 min of common carotid artery occlusion (CCAO). (3) CCAO, rats with CCAO only. (4) Sham group, rats with mock CCAO. (5) Untreated rats. *∗*, significant when compared with the control group; ANOVA test and post hoc Dunnett's test. †, significant when compared between groups; unpaired Student's *t*-test. *P* < 0.05.

CCR2 mRNA levels in the sham group decreased overtime (Figure 2(a)). CCAO decreased CCR2 mRNA levels by 47 ± 3% at 96 h after reperfusion with respect to those of time 0 (Figure 2(a)). The subacute administration of zinc increased CCR2 mRNA levels by 291 ± 92% at −48 h and by 309 ± 63% at 24 h in control rats (Figure 2(b)). CCAO produced upregulation of CCR2 mRNA levels by 330 ± 61% at 4 h, by 207 ± 56% at 8 h, and by 30 ± 6% 36 h after reperfusion only in rats treated with zinc (Figure 2(b)).

CCAO increased CCR2 protein levels by 66 ± 4% at 8 h and by 75 ± 43% at 168 h after reperfusion at time 0 and in comparison to the sham group (Figure 2(c)). The subacute administration of zinc increased CCR2 protein levels at time 0 by 146 ± 27%, by 46 ± 4% at 8 h, and by 32 ± 0.2% at 36 h after reperfusion (Figure 2(d)).

**Figure 3:**
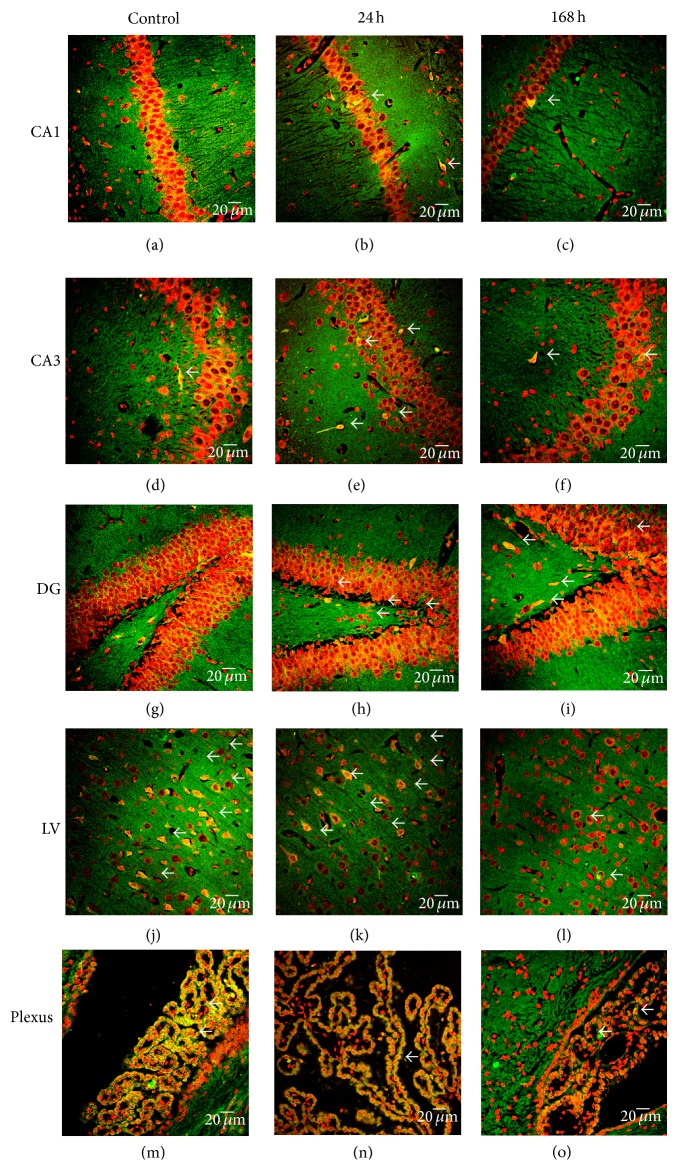
Effect of common carotid artery occlusion on the immunoreactivity against CCR2. Representative micrographs of CCR2 immunofluorescence ((a) to (o)) using a rabbit antibody against CCR2 and a goat antibody anti-rabbit IgG conjugated with fluorescein isothiocyanate (green color). The cerebral region was identified by propidium iodide (red color) counterstaining. CA, Cornu Ammonis, and DG, dentate gyrus, of the hippocampus. LV, layer V of the cerebral cortex. Plexus, choroid plexus. Values are the mean ± SEM from 3 rats in each experimental condition.

**Figure 4:**
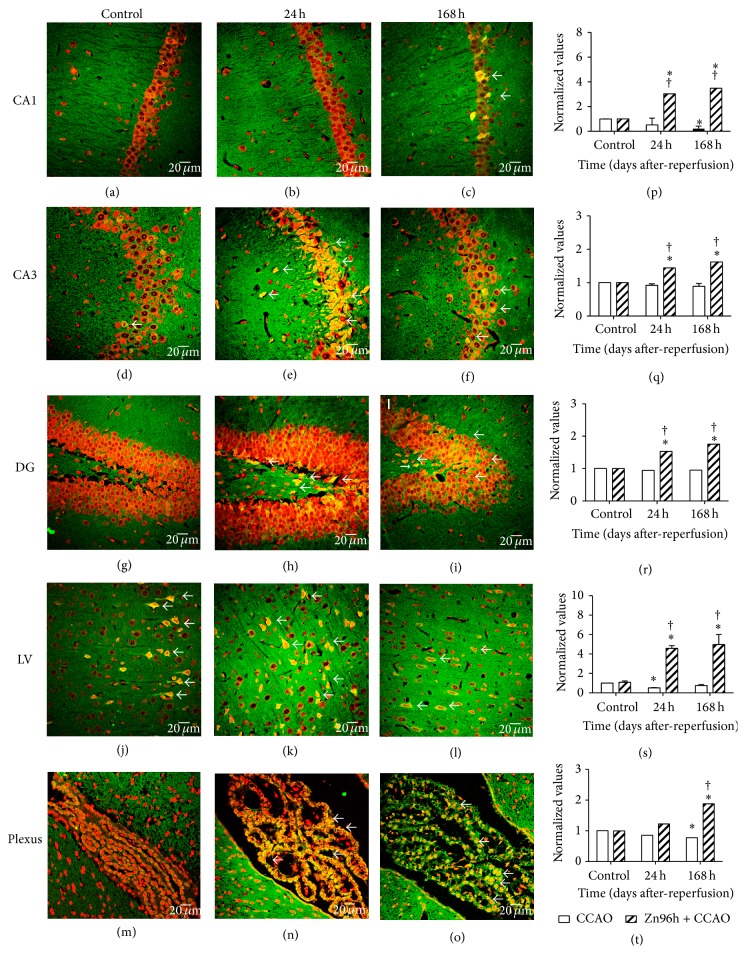
Effect of the subacute administration of zinc on CCR2 immunoreactivity after CCAO. Representative micrographs of CCR2 immunofluorescence ((a) to (o)) using a rabbit antibody against CCR2 and a goat antibody anti-rabbit IgG conjugated with fluorescein isothiocyanate (green color). The cerebral region was identified with propidium iodide (red color) counterstaining. Graphs ((p), (q), (r), (s), and (t)) show the normalized values of CCR2-IR with respect to control IR for CCAO (Figures 3(a) to 3(o)) and subacute administration of zinc ((a) to (o)). The IR was measured using ImageJ 1.45 of the National Institute of Health. CA, Cornu Ammonis, and DG, dentate gyrus, of the hippocampus. LV, layer V of the cerebral cortex. Plexus, choroid plexus. Values are expressed as the mean ± SEM from 3 rats for each experimental condition. *∗*, significant when compared with the control group; ANOVA test and post hoc Dunnett's test. †, significant when compared between groups; unpaired Student's *t*-test. *P* < 0.05.

The regional localization of CCR2 was explored using indirect immunofluorescence and propidium iodide counterstaining. CCR2 immunoreactivity (IR) was mainly localized in the granular zone of hippocampus (Figures 3 and 4) and in the pyramidal cells of layer III (data not shown) and V of cerebral cortex (Figures 3 and 4). The net intensity of CCR2 immunofluorescence was plotted (number of pixels per area) comparing each region with CCAO of Figures 3(a)–3(o) against subacute administration of zinc after CCAO in Figures 4(a)–4(o), and these are showed in the graphs of Figure 4 ((p), (q), (r), (s), and (t)). In the CA1 region, CCAO (Figure 3(c)) caused a significant decrease in CCR2-IR by 79 ± 15% at 168 h after reperfusion (Figure 4(p)). In the cerebral cortex (Figure 3(k)), the decrease was 46 ± 2% at 24 h after reperfusion (Figure 4(s)) and in choroid plexus (Figure 3(o)); CCAO caused a decrease of CCR2-IR by 31 ± 0.5% at 168 h after reperfusion (Figure 4(t)). CCAO caused the loss of granular cell cytoarchitecture of CA1, CA3, and DG regions (Figures 3(a)–3(i)). Whereas the subacute administration of zinc caused a significant increase of CCR2 IR in the CA1 after CCAO at 24 h (Figure 4(b)) by 202 ± 5% and at 168 h after reperfusion (Figure 4(c)) by 248 ± 3% as shown in the graph (Figure 4(p)). The net intensity in CA3 after CCAO at 24 h (Figure 4(e)) increased 44 ± 2% and at 168 h after reperfusion (Figure 4(f)) by 62 ± 2% as shown in the graph (Figure 4(q)). In the DG (Figures 4(h) and 4(i)), CCR2 IR increased by 53 ± 3% at 24 h and by 75 ± 2% at 168 h after reperfusion (Figure 4(r)). In layer V of cerebral cortex (Figures 4(k) and 4(l)), CCR2 IR increased by 337 ± 5% at 24 h and by 324 ± 7% at 168 h after reperfusion (Figure 4(s)). In the choroid plexus (Figure 4(o)), CCR2 IR increased by 88 ± 2% at 168 h after reperfusion (Figure 4(t)). In choroid plexus, the CCR2 IR increased at 168 h after reperfusion (Figure 4(o)) by 88 ± 2% as shown in the graph (Figure 4(t)).

**Figure 5:**
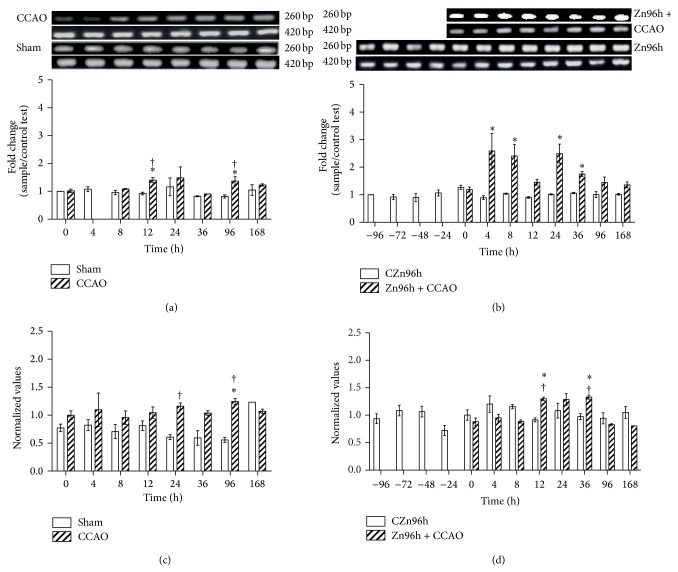
The subacute administration effect of zinc on FGF2 expression levels after hypoxia-ischemia in the rat. RT-PCR was used to determine mRNA levels of FGF2 (260 bp) and GA3PDH (420 bp). ((a) and (b)) Showing representative photographs of ethidium-bromide-stained RT-PCR products fractionated on 2% agarose gel and the respective densitometry analysis. ((c) and (d)) Showing the FGF2 protein levels measured using ELISA. Each value represents the mean ± SEM of 5 independent experiments made in triplicate. (1) Zn96h, rats injected with ZnCl_2_ (one dose every 24 h during 4 days). (2) Zn96h + CCAO, rats treated with zinc before 10 min common carotid artery occlusion (CCAO). (3) CCAO, rats with CCAO only. (4) Sham group, rats with mock CCAO. (5) Untreated rats. *∗*, significant when compared with the control group; ANOVA test and post hoc Dunnett's test. †, significant when compared between groups; unpaired Student's *t*-test. *P* < 0.05.

**Figure 6:**
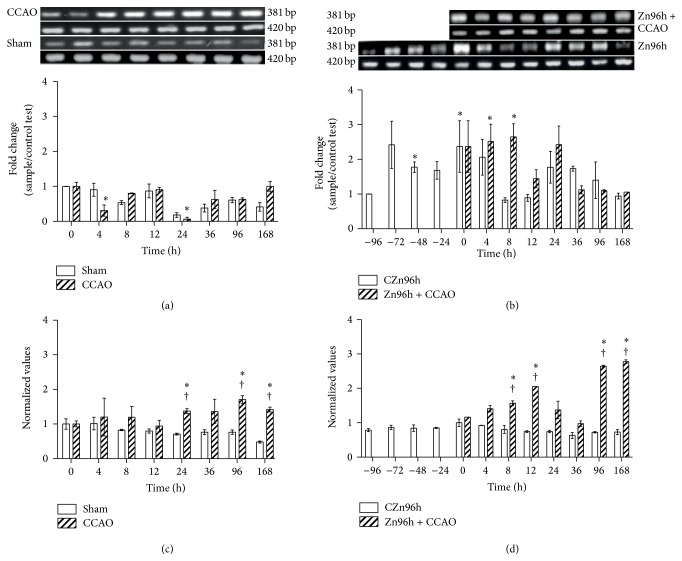
The subacute administration effect of zinc on levels of IGF-1 expression after hypoxia-ischemia in rats. RT-PCR was used to determine mRNA levels of IGF-1 (381 bp) and GA3PDH (420 bp). ((a) and (b)) Showing representative photographs of ethidium-bromide-stained RT-PCR products fractionated on 2% agarose gel and the respective densitometry analysis. ((c) and (d)) Showing the IGF-1 protein levels measured using ELISA. Each value represents the mean ± SEM of 5 independent experiments in triplicate. (1) Zn96h, rats injected with ZnCl_2_ (one dose every 24 h during 4 days). (2) Zn96h + CCAO, rats treated with zinc before 10 min of common carotid artery occlusion (CCAO). (3) CCAO, rats with CCAO only. (4) Sham group, rats with mock CCAO. (5) Untreated rats. *∗*, significant when compared with the control group; ANOVA test and post hoc Dunnett's test. †, significant when compared between groups; unpaired Student's *t*-test. *P* < 0.05.

RT-PCR and ELISA were used to identify whether the subacute administration of zinc modifies the expression of FGF2 (Figure 5) and IGF1 (Figure 6) after CCAO. An increase in FGF2 mRNA levels by 40 ± 9% at 12 h and by 37 ± 6% at 96 h was detected after reperfusion (Figure 5(a)). The subacute administration of zinc caused a further increase in FGF2 mRNA by 214 ± 55% at 4 h, 141 ± 42% at 8 h, 149 ± 35% at 24 h, and 75 ± 9% at 36 h after reperfusion (Figure 5(b)).

CCAO also increased FGF2 protein levels by 25 ± 6% at 96 h after reperfusion when compared to the control (time 0; Figure 5(c)). The subacute administration of only zinc increased FGF2 protein levels by 33 ± 13% at 4 h with respect to the last administration (Figure 5(d)), while CCAO caused two further increases, first by 30 ± 3% in the early phase (12 h) and the second by 33 ± 4% in the late phase (36 h) after reperfusion in rats treated with zinc (Figure 5(d)).

CCAO decreased IGF-1 mRNA levels by 69 ± 16% at 4 h and by 94 ± 6% at 24 h after reperfusion when compared to the control at time 0 (Figure 6(a)). A significant decrease of 82 ± 7% in IGF1 mRNA levels was observed at 24 h after mock surgery (Figure 6(a), white bars). The subacute administration of only zinc increased IGF1 mRNA levels by 77% ±15% before time 0 (Figure 5(b)) from −48 h until time 0 and these values were normalized to the basal values after time 0. CCAO maintained the zinc-induced upregulation until 8 h after reperfusion in rats treated with zinc (Figure 6(b)).

CCAO increased IGF1 protein levels by 37 ± 7% at 24 h, 71 ± 11% at 96 h, and 42 ± 7% at 168 h after reperfusion when compared with the control at time 0 (Figure 6(c)), whereas the subacute administration of zinc increased IGF-1 levels by 41 ± 9% at 8 h, with a maximum of 106 ± 0.1% at 12 h in the early phase after CCAO, and by 164 ± 3% at 96 h and 178 ± 5% at the 168 h after reperfusion (Figure 6(d)).

**Figure 7:**
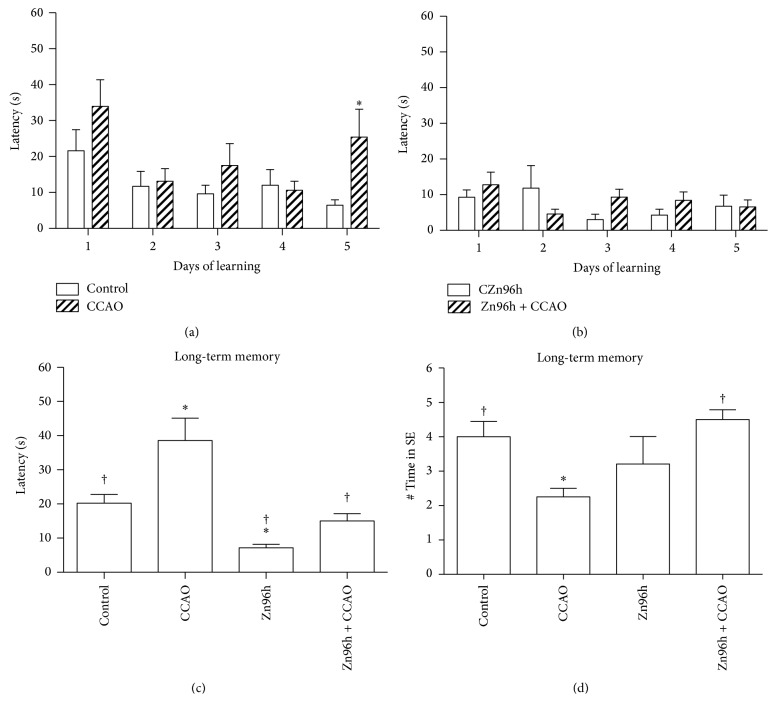
The effect of subacute administration of zinc on learning and long-term memory after hypoxia-ischemia in rats. ((a) and (b)) Graphs showing the latency to reach the escape platform in the fourth event (eastern quadrant) of a daily evaluation for five days in the Morris Water Maze (*n* = 10 rats per group). (c) Graphs showing the latency determined on day 7 after the learning training, that is, on day 12 after reperfusion (*n* = 5 rats per group). (d) Graphs showing the number of times in southeast (# Time in SE) at which rats pass by the platform location. Evaluations were made on day 7 after the learning training. The values are the mean ± SEM. *∗*, significant when compared with the control group ANOVA test and post hoc Dunnett's test, *P* < 0.05. †, significant when compared with the CCAO group; unpaired Student's *t*-test. *P* < 0.05.

To evaluate whether the subacute administration of zinc prevents the CCAO-induced neuronal damage in the hippocampus, spatial reference learning, and memory was assessed using Morris Water Maze. CCAO increased the learning latency by 291 ± 119% on day 5 with respect to the untreated group (Figure 7(a)). Interestingly, the subacute administration of zinc alone significantly decreased the learning latency (Figure 7(b)) compared to the untreated group (Figure 7(a)), suggesting that the learning was improved. Furthermore, the subacute administration of zinc prevented the CCAO-induced increase on day 5 (Figure 7(b)). CCAO also increased the latency by 43 ± 6% on day 7 after the learning training, which was on day 12 after reperfusion (Figure 7(c)). Remarkably, the subacute administration of zinc alone decreased the latency by 64.4 ± 4.8% when compared with the untreated group and prevented the hypoxia-ischemia-induced increase on day 12 after reperfusion (Figure 7(c)). In addition, the number of times at which rats pass by the platform location was decreased by 50% in the CCAO group as compared with the untreated control group. Such decrease was prevented by the prophylactic subacute administration of zinc (Figure 7(d)), thus suggesting retention of long-term memory.

## 4. Discussion

Our results show that the prophylactic subacute administration of zinc causes a neuroprotective effect in the hippocampus and cerebral cortex, by increasing the expression of neurotrophic factors CCL2, CCR2, FGF2, and IGF-1 in the early and late phases after transient hypoxia-ischemia process and preventing the loss of the long-term in spatial reference memory as evaluated in the Morris Water Maze. Supporting the neuroprotective effect, previous studies have demonstrated that zinc decreases the nitrosative stress, inflammatory cytokines, and cell death after hypoxia-ischemia process and atherosclerosis [1, 3, 16].

The subacute administration of zinc induced CCL2 expression in the early phase after reperfusion. A mechanism that might mediate the upregulation of CCL2 is the activation of the zinc finger transcription factor ZXDC in astrocytes and microglial cells, known to be antigen-presenting cells in the central nervous system [58]. This suggestion is supported by the finding that the overexpression of zinc finger transcription factor ZXDC induces CCL2 gene expression in antigen presenting cells, such as the human leukemic monoblast cell line U937 [58]. Because CCL2 plays a neuroprotective role during an ischemic preconditioning and postconditioning process [5, 40, 41], zinc finger transcription factor ZXDC might mediate CCL2-induced neuroprotection. Moreover, several references support the upregulation of CCL2 through HuR proteins, miRNAs, and inflammatory cytokines (IL-17 and TNF*α*), which promote stability of CCL2 mRNA after their binding to ARE's regions [59–61]. These mechanisms might also mediate CCL2-induced neuroprotection administered when zinc is administered prior to the ischemic event.

Several studies support that the increase in CCL2 levels promotes the inflammatory process: such an effect has been reported to be harmful [36–39, 62, 63]. In addition, CCL2 is widely recognized to be a major component of chronic inflammation associated with a variety of diseases including obesity-associated type 2 diabetes and cardiovascular diseases [64, 65]. Some proposed mechanisms of CCL2 induced-increase in inflammation are the endoplasmic reticulum stress and autophagy [62].

In contrast, other studies have shown that CCL2 plays a neuroprotective role [28, 29, 35, 40, 41]. Accordingly, CCL2 upregulation after ischemic preconditioning [40] and postconditioning prevents ischemia/reperfusion-induced cerebral injury [41]. In addition, the subacute administration of zinc also caused the expression of CCR2 in granular cells of the hippocampus and pyramidal cells of the cerebral cortex in the early and late phases after CCAO, thus suggesting a reinforcement of the neuroprotective effect. In support of this suggestion, there are evidences that CCL2/CCR2 expressed in neurons during an ischemic preconditioning and postconditioning process attenuates the reperfusion-induced injury and reduces the release of systemic proinflammatory cytokines [5, 40, 41]. Moreover, CCL2/CCR2 has been reported to promote the recruitment of neural stem cells [31, 66]. The overexpression of CCL2 together with other chemokines such as CCL3 and CXCL1 can redirect the precursor cell migration into a nonneurogenic region [67, 68]. Thus, zinc through CCL2 and other chemokines might contribute together with neurotrophic factors in the functional restoration in animal models of neural injury and neurodegeneration.

We found that the subacute administration of zinc also increases the expression of FGF2 in the early phase and IGF-1 in the early and late phases after hypoxia-ischemia process. The increase in these growth factors might be accounted by the increased levels of CCL2 [29, 30, 69] or by the effect of zinc through other unknown mechanisms. For instance, zinc supplement is able to increase the protein levels of IGF-1 and IGFBP3 in plasma in children [13] and FGF2 expression by myeloid zinc finger protein-1 (MZF-1) in astrocytes [70]. In addition, the presence of zinc potentiates the stimulation by FGF2 and FGF1 of the proliferation in cultured vascular smooth muscle cells [71].

An increasing number of studies sustain that FGF2 and IGF-1 participate in neurogenesis, neurodifferentiation, and neuron survival [30]. On this basis, we propose that the presence of FGF2 and IGF-1 in the early phase might activate the proliferation of progenitor cells and that the IGF-1 in the late phase and in the absence of FGF2 might promote the differentiation of neural precursor neurons [31].

FGF2 and IGF-1 also have an anti-inflammatory effect, by reducing the blood-brain barrier permeability at 4 h after stroke, suppressing the serum levels of cytokines including IL-6, IL-10, and TNF-*α* [72, 73], and stimulating DNA repair, metabolic homeostasis, cytoskeletal stability, and cholesterol biosynthesis [74]. In addition, IGF-1 is known to decrease the response of microglia to ischemic stroke and lipopolysaccharides [46]. Moreover, any form of administration of IGF-1 has shown a neuroprotective effect for the treatment of acute ischemic stroke [48]. FGF2 also exhibits an antioxidant effect on the redox system and reduces the oxidative stress induced by bisphosphonate [74]. Therefore, we propose that the overexpression of FGF2 and IGF-1 induced by the subacute administration of zinc might play an antioxidant, anti-inflammatory, and neurogenic role after a hypoxia-ischemia process.

Although there are various methods to measure the functionality of hippocampus, the Morris Water Maze has the advantage that it strongly correlates the measurements of spatial reference memory with the hippocampal synaptic activity and NMDA receptor function [75, 76]. Furthermore, the task in the Morris Water Maze requires only the stimulus of escape from water. This represents an additional advantage on radial arm maze or T-maze that requires deprivation of food or water [77]. It has been reported that zinc supplementation is an effective treatment option for improving cognitive deficits [78, 79], but not for improving motor activity [79] in rats evaluated using the Morris Water Maze. Using this apparatus, a recent report shows that nanoZnO treatment improves the spatial memory and the synaptic plasticity, which was assessed by measuring the long-term potentiation (LTP) in the DG [80]. Our results using this behavioral test showed that the subacute zinc administration improves the long-term memory in control rats and prevents the CCAO-induced loss of spatial reference memory. An increased zinc store in synaptic vesicles of hippocampal mossy fibers might account for the beneficial effect of zinc administration on spatial reference memory because it is widely known that the corelease of zinc and glutamate from the mossy fibers favors the learning and memory [81]. In addition, an increase in FGF2 and IGF-1 expression reported here might contribute to synaptic plasticity, which would consolidate the learning and memory after CCAO [82, 83]. Other beneficial effects of FGF2 and IGF-1 involved in the subacute administration of zinc might be the stimulation of neuron survival and neurogenesis in the subventricular zone and the subgranular zone of dentate gyrus, since these events are triggered in adult rats following cerebral ischemia [47, 50].

## 5. Conclusion

The main outcome of this work is that the subacute administration of zinc increases the expression of CCL2, CCR2, and growth factors (FGF2 and IGF-1) as well as preventing the loss of memory in the rats after transient hypoxia-ischemia. This latter result suggests that the prophylactic administration of zinc exerts a neuroprotective effect in the cerebral hypoxia-ischemia model, possibly inducing neuronal plasticity.

## Figures and Tables

**Table 1:** List of primers to amplify chemokine and growth factors.

Sequence accession in NCBI		Sequence	Position	Amplicon
NM_019305.2	FGF2-f	GGAGTTGTGTCCATCAAGG	736–754	260
	FGF2-r	CCAATGTCTGCTAAGAGCTG	996–977	

NM_001082477.2	IGF1-f	TCGTCTTCACATCTCTTCTACC	64–85	381
	IGF1-r	GTGTACTTCCTTTCCTTCTCCT	445–424	

NM_031530.1	CCL2-f	GGAGAACTACAAGAGAATCACC	218–239	323
	CCL2-r	GCATCACATTCCAAATCACAC	541–521	

NM_021866.1	CCR2-f	CAGGGCTTTATCACATTGGG	388–407	311
	CCR2-r	AGATGACCATGACAAGTAGCG	699–679	

AF_10686	GA3PDH-f	AAAACAGTCCATGCCATCAC	360–380	420
	GA3PDH-r	TCCACCACCCTGTTGCTGTA	780–759	

The primers were designed using Perl Primer V.1.1.19-1 (graphical design of primer for PCR) and their identity was analyzed using BLAST of the NCBI.
